# Human Bone Marrow versus Adipose-Derived Stem Cells: Influence of Donor Characteristics on Expandability and Implications for Osteogenic Ex Vivo BMP-2 Regional Gene Therapy

**DOI:** 10.1155/2023/8061890

**Published:** 2023-08-14

**Authors:** Cory K. Mayfield, Elizabeth Lechtholz-Zey, Mina Ayad, Osamu Sugiyama, Jay R. Lieberman

**Affiliations:** Keck School of Medicine of USC, Department of Orthopaedic Surgery, Los Angeles, USA

## Abstract

Novel treatment strategies for segmental bone loss in orthopaedic surgery remain under investigation. Regional gene therapy that involves transduction of mesenchymal stem cells with a lentiviral vector that expresses BMP-2 has gained particular interest as this strategy provides osteogenic and osteoinductive factors for bone growth. In particular, transduced adipose-derived stems cells (ASCs) and bone marrow-derived stem cells (BMSCs) have emerged as the leading candidates for the treatment of segmental defects in preclinical models. The aim of the present study was to evaluate the influence of demographic information on *in vitro* growth characteristics and bone morphogenetic protein-2 production following lentiviral transduction in a large cohort of human donors. We further sought to assess the effects of ASC harvest site on cell yield and growth characteristics. We evaluated a total of 187 human donors (124 adipose harvests and 63 bone marrow aspirates) in our cohort. We found that across all donors, ASCs demonstrated favorable growth characteristics and could be cultured *in vitro* more reliably than BMSCs regardless of patient-related factors. Furthermore, we noted that following lentiviral transduction, ASCs produced significantly higher levels of BMP-2 compared to BMSCs. Lastly, despite higher initial cell yields from lipoaspirate, posttransduction BMP-2 production was less than that of infrapatellar fat pad samples. These results support the continued investigation of ASCs as a cellular delivery vehicle for regional gene therapy to deliver osteoinductive proteins to specific anatomic bone repair sites.

## 1. Introduction

Significant bone loss is a common and complex challenge that may occur in the setting of acute trauma, fracture non-union, and revision joint replacement. To promote the bone healing required to effectively address these clinical challenges, four essential elements must be present: an appropriate osteoinductive signal; stem cells that can respond to the osteoinductive signal; an osteoconductive scaffold capable of supporting the bone formation process; and sufficient blood supply to deliver cytokines to the repair site [1]. The use of autograft has long been the gold standard for treatment in these cases; however, this technique is limited in supply and by considerable donor site morbidity [2, 3]. The challenge associated with the treatment of large bone defects is the lack of responding cells within a stringent biological environment that may have compromised vascularity and the requirement of sustained release of osteoinductive proteins. Due to these challenges, bone tissue engineering with mesenchymal stem cells (MSCs) has emerged as an alternative approach to addressing this clinical problem.

Although MSCs have shown promise in tissue engineering applications, their use alone has proven insufficient in the treatment of bone defects without augmentation with osteoinductive growth factors or proteins [4, 5]. The concomitant use of gene therapy strategies with MSCs has emerged as a potential method to further augment bone regeneration through sustained upregulation of osteoinductive growth factors. For example, *ex vivo* regional delivery of bone morphogenetic protein-2 (BMP-2) using a lentiviral vector results in sustained release of BMP-2 that can promote a substantial bone healing response [6, 7]. Because there are a variety of MSCs that form the foundation of bone tissue engineering, the choice of which type of MSC to use is an important one [8, 9]. Bone marrow-derived stem cells (BMSCs) have historically been the choice for researchers [10]; however, the harvest of bone marrow is an invasive procedure, often limited by low stem cell yields contained within the aspirate. A particularly interesting alternative to BMSCs is adipose-derived stem cells (ASCs) due to the ease of their harvest, which can be acquired from a variety of anatomical locations through minimally invasive procedures, such as liposuction and knee arthroscopy [11]. Other potential advantages include high aspirate cell yields, reliable *in vitro* proliferation, and sustained BMP-2 expression following *ex vivo* gene therapy transduction [12].

The uses of ASCs for bone tissue engineering, and comparisons of their *in vitro* characteristics and osteogenic potential relative to BMSCs, have been investigated in non-human models and small human cohorts [12–14]. The current literature is limited by the lack of data assessing the clinical potential of both human adipose and bone marrow-derived stem cells from a substantial patient cohort. Therefore, the purpose of this study is to further characterize the differences in the *in vitro* expansion potential and lentiviral-transduced BMP-2 production of ASCs and BMSCs from a large cohort of human patients. Furthermore, we aimed to assess the impact of patient-related factors as well as harvest location and technique on cellular expansion. We hypothesized that the ASCs would exhibit superior *in vitro* expansion characteristics and lentiviral-mediated BMP-2 production, providing further evidence that they may present the optimal source of mesenchymal stem cells in bone tissue engineering applications for addressing critical-sized bone defects.

## 2. Materials and Methods

### 2.1. Isolation of Human Bone Marrow Cells

Under IRB approval (IRB # HS-17-00860), human bone marrow cells were harvested from the femoral intramedullary canals of patients who underwent total hip arthroplasty at our institution. We recorded patients' sex, age, race, body mass index, preoperative serum albumin, and hemoglobin A1c. The bone marrow is typically cleared from the canals prior to insertion of the femoral implant, and thus a strategy to collect this material was utilized [1]. Bone marrow (BM) was combined with PBS (Lonza, Basel, CH) and centrifuged at 400 g for 5 minutes. The mononuclear cells were then isolated from the raw BM using Histopaque 1077 (Sigma-Aldrich, St. Louis, MO), washed, and incubated with ACK Lysing Buffer (Lonza, Basel, CH) for one minute to remove the remaining red blood cells. The cell pellet was resuspended in Dulbecco's modified eagle medium (DMEM; Corning Mediatech, Manassas, VA) with 10% fetal bovine serum (FBS; VWR, Radnor, PA) and supplemented with an antibiotic mix containing 100 unit/ml penicillin, 100 *µ*g/mL streptomycin, and 250 ng/mL amphotericin B (Lonza, Basel, CH).

### 2.2. Isolation of Human Adipose-Derived Stem Cells

Under IRB approval, we collected infrapatellar fat pads from patients undergoing total knee arthroplasty (TKA) and arthroscopic knee surgery (AKS) at our institution, as well as lipoaspirate samples from abdominal or buttock liposuction. Liposuction refers to the surgical procedure utilized to harvest adipose tissue while lipoaspirate refers to the collected material containing adipose-derived stem cells [15]. The infrapatellar fat pads and lipoaspirate were processed using a previously described protocol [1, 7]. In brief, adipose samples from both fat pads and lipoaspirates were mechanically digested into 3 mm pieces, which were washed with PBS (Lonza, Basel, CH) and centrifuged at 400 g for 5 minutes. Samples were then enzymatically digested with 0.1% collagenase (type 1; Sigma-Aldrich, St. Louis, MO) for 90 minutes at 37°C. To neutralize the enzymatic activity, a 1 : 1 ratio of DMEM was added to each sample. After subsequent centrifugation at 1200g for 10 minutes, the stromal vascular fraction (SVF) was identified and strained through a 100 *µ*m filter. The SVF cells were resuspended in DMEM containing 10% FBS and the antibiotic mix as above.

### 2.3. Cell Culture

Mononuclear cells isolated from BM and the SVF isolated from adipose samples were counted with a cytometer using trypan blue. The protocol for the isolation of the cell population of interest has been previously characterized by our lab [1, 13], so additional steps to confirm the cell populations were not performed for this study. BM mononuclear cells were plated at a density of 3-4 × 10^7^ cells per 10 cm dish, and the SVF containing adipose-derived stem cells (ASCs) were plated at a density of 2-3 × 10^6^ cells/dish.

The plated cells were maintained in a humidified environment at 37°C in 5% CO_2_ throughout culture expansion. Cell media were changed every 3 days to remove non-adherent cells and other contaminants. BMSCs were cultured for two weeks before they were designated at passage 1, while ASCs reached passage 1 after 1 week in culture. At each passage, confluent cells were trypsinized (Thermo Fisher, Waltham, MA), counted, and replated at their respective densities.

### 2.4. Transduction with Lentivirus

Lentiviral transduction of BMSCs and ASCs was performed using a two-step transcriptional amplification (TSTA) system, as described in a previous protocol [1, 13]. In brief, TSTA consists of the GAL4-VP16 transactivator vector and the G5 transgene expression vector encoding the BMP-2 cDNA. Lentiviral stock was generated by transfecting 293T cells (American Type Culture Collection, Manassas, VA) as previously described [13].

After verification of lentiviral vector titers using a previously described protocol [1], BMSCs and ASCs were transduced after they reached either passage 3 or passage 5, only. Cells were plated at a density of 1 × 10^6^ cells/10 cm dish the day prior to transduction. Using the LV-RhMLV-GAL4-VP16 promoter and LV-G5-BMP cDNA each at a multiplicity of infection (MOI) of 3, cells were transduced in the presence of 8 *µ*g/mL Polybrene. After 16 hours in culture in the presence of transduction media, plates were washed with PBS and allowed to culture in DMEM + 10% FBS for an additional 24 hours before the final harvest to quantify *in vitro* BMP-2 production.

### 2.5. Quantification of BMP-2 Production *In Vitro*

After culture, the supernatant was collected from plates containing ASC/lentiviral-two-step transcriptional amplification-bone morphogenetic protein-2 (LV-TSTA-BMP-2) and BMSC/LV-TSTA-BMP-2. This supernatant was used to quantify BMP-2 production over a 24-hour period using ELISA (Quantikine, R&D systems, Minneapolis, MN). Samples were run in duplicate and reported as an average in nanograms of BMP-2 per 1 × 10^6^ cells per day. Based on internal experiments, cell counts do not significantly change after these 40 hours in culture, so the BMP-2 production over the 24 hour period obtained via ELISA is consistently based on the 1 × 10^6^ plated cells.

### 2.6. Population Doubling Time

Population doubling time (PDT) was determined based on generation time (number of days between subsequent passages) and cell counts obtained by automated cell counter cytometry with trypan blue. PDT was calculated for both BMSCs and ASCs between all passages. PDT was calculated using the doubling time/exponential growth equation where *t*_2_ − *t*_1_ = time in days from, for example, P2 to P3, and *q*_2_/*q*_1_ represent cell numbers at, for example, P3 and P2, respectively [1].

(1) $$\begin{array}{c} T_{d}=\left(t_{2}-t_{1}\right)\cdot \frac{\ln\left(2\right)}{In\left(Q_{2}/Q_{2}\right)}\text{.} \end{array}$$

### 2.7. Statistical Analysis

Data were collected and gathered into Microsoft Excel (Microsoft Corporation, Redmond, WA). Statistical analysis was performed using STATA17BE (StataCorp LLC, College Station, TX). Descriptive statistics were employed with a Mann–Whitney U test for continuous variables and a chi-squared analysis for categorical variables. A Pearson correlation analysis was performed to analyze the relationship between BMP-2 production and age. Significance was set at *p* < 0.05.

## 3. Results

### 3.1. Patient Demographics and Sample Characteristics

**Figure 1:**
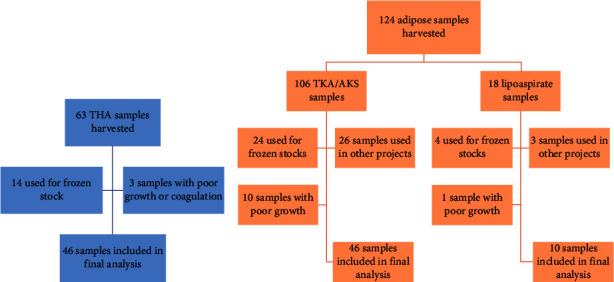
Flowchart depicting how samples were distributed given retrospective nature of this project. 46 samples obtained via total hip arthroplasty (THA) were ultimately included. 46 infrapatellar fat pad samples obtained via total knee arthroplasty (TKA) or arthroscopic knee surgery (AKS) were ultimately included. 10 lipoaspirate samples were included in the final analysis.

A total of 63 patients underwent total hip arthroplasty (THA) at our institution for isolation of human bone marrow-derived stem cells. From the cohort of patients who underwent THA, 14 samples were used to create frozen stocks and were thus excluded from analysis. Two samples were discarded due to poor growth, and one sample was excluded due to coagulation prior to processing. A total of 46 THA samples were used in the final analysis. Infrapatellar fat pads were harvested from 106 patients that underwent total knee arthroplasty (TKA) or arthroscopic knee surgery (AKS) (93 and 13 patients, respectively). From the TKA/AKS group, 26 samples underwent final culture through passage 3, while a total of 24 samples were used to create frozen stocks. Ten samples exhibited poor growth prior to passage 3 and were discarded, leaving a total of 46 samples for analysis. In addition, 18 patients underwent liposuction, for the isolation of human adipose-derived stem cells (Figure 1). Demographic data for all patients are shown in Table 1.

### 3.2. Assessment of Cell Proliferation and Yield

#### 3.2.1. Bone Marrow Aspirate versus Infrapatellar Fat Pad

**Figure 2:**
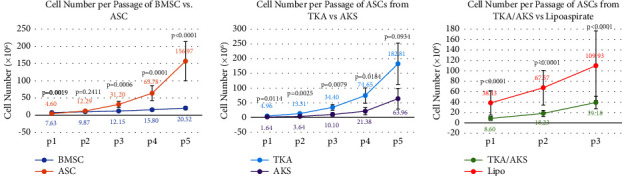
Comparisons of cell counts at each passage between groups. Mann–Whitney U test was used to determine statistical significance between BMSCs and ASCs at each passage. (a) Comparison between bone marrow-derived stem cells and adipose-derived stem cells. Samples per group: P1: 62 BMSCs, 101 ASCs; P2: 58 BMSCs, 95 ASCs; P3: 51 BMSCs, 76 ASCs; P4: 45 BMSCs, 49 ASCs; P5: 38 BMSCs, 46 ASCs. (b) Comparison of adipose-derived stem cells harvested during total knee arthroplasty (TKA) versus arthroscopic knee surgery (AKS). Samples per group: P1: 90 TKA, 11 AKS; P2: 84 TKA, 10 AKS; P3: 66 TKA, 10 AKS; P4: 39 TKA, 10 AKS; P5: 36 TKA, 10 AKS. (c) Comparison of adipose-derived stem cells harvested during TKA/AKS versus liposuction. Samples per group: P1: 101 TKA/AKS, 15 lipo; P2: 95 TKA/AKS, 13 lipo; P3: 76 TKA/AKS, 10 lipo.

Cell yield and proliferation data are shown in Table 2. There was a significant difference in the total isolated nucleated cell count between infrapatellar fat pads and bone marrow aspirates (*p* < 0.0001). Additionally, the cell yield per 1 mL of tissue was significantly greater in the THA group than in the TKA/AKS group (*p* < 0.0001). When comparing cell yield/mL of harvested tissue both within the THA group and within the TKA/AKS group, there were no differences by sex or between patients ≤60 years old and those >60 years old. A comparison of cell counts between the BMSCs and ASCs showed significant differences at P1 and P3 through P5 (*p* < 0.001) (Figure 2(a)). Lastly, population doubling times were consistently shorter for ACSs than for BMSCs at each passage (Table 3).

#### 3.2.2. Growth Comparisons

Both sample types were separated into groups based on whether they exhibited normal (NG) or slow/poor growth (PG) characteristics during culture expansion. 38 of 46 BMSC samples were successfully expanded from P3 to P5 (82.6%), with the other eight exhibiting poor or declining growth. For this analysis, we also included samples that were used to create frozen stocks after P3 (*n* = 5) in the “normal growth” group. 46 infrapatellar fat pad samples were cultured from P3 to P5 with all of these samples reaching passage 5. For both BMSCs and ASCs, there were no significant differences in age, gender, or harvested tissue volume between cohorts. BMSCs also showed no differences in total isolated nuclear cell count or cell yield/mL between the groups. However, samples from the ASC NG group yielded a much greater stromal vascular fraction than the ASC PG group (*p* = 0.015), as well as a significantly greater cell yield/mL (*p* = 0.009). Additionally, there were no significant differences in patients' BMI, serum albumin, or preoperative HbA1c for both BMSCs and ASCs (Table 4).

#### 3.2.3. Total Knee Arthroplasty versus Arthroscopic Knee Surgery

To compare open versus arthroscopic infrapatellar fat pad harvest, we conducted further analysis into the characteristics of cells obtained from the infrapatellar fat pad by TKA versus AKS (Table 5). Patients who underwent TKA tended to be much older (64.67 ± 8.24 years) than those who underwent AKS (37.62 ± 14.17) (*p* < 0.0001) with no significant difference in sex. The stromal vascular fraction and cell yield/mL were greater in TKA than in AKS (*p* < 0.0001 and *p*=0.012, respectively). When cell counts were assessed at five passages, the TKA group exhibited significantly greater cell yield than the AKS group from P1 to P4 (Figure 2(b)). At P5, there was no significant difference.

#### 3.2.4. Infrapatellar Fat Pad versus Lipoaspirate

We additionally sought to compare the effects of harvest site by comparing the TKA/AKS cohort to the lipoaspirate cohort (Table 5). Patients who underwent TKA/AKS tended to be much older (64.67 ± 8.24 years) than those who underwent liposuction (39.56 ± 8.51 years) (*p* < 0.0001), and 100% of liposuction patients were female compared with 50.54% of TKA/AKS patients (*p* < 0.0001). The SVF of lipoaspirate was much greater than that of infrapatellar fat pads (*p* < 0.0001). However, there was a greater cell yield/mL in the TKA/AKS group (*p* < 0.0001). Cell counts were consistently greater in the lipoaspirate group than in the TKA/AKS group at each passage (Figure 2(c)).

### 3.3. *In Vitro* BMP-2 Production by ASCs and BMSCs Transduced with LV-TSTA-BMP-2 and LV-GVP-BMP-2

**Figure 3:**
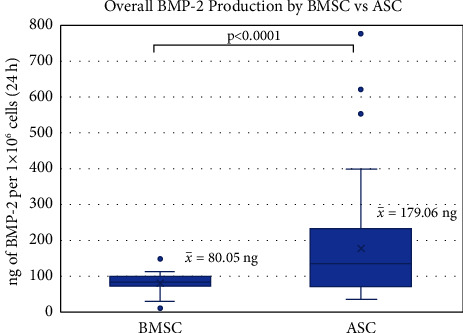
Comparison of BMP-2 production between transduced BMSCs and ASCs across all transduction methods. 54 BMSC samples and 59 ASC samples were included in this analysis. Mann–Whitney U test was used to determine statistical significance.

**Figure 4:**
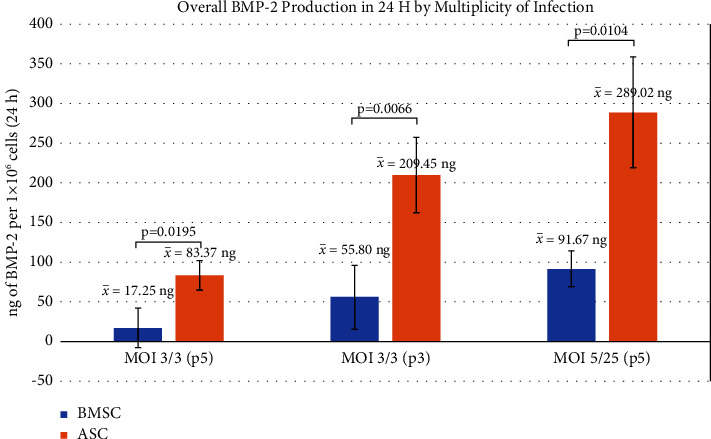
Comparison of BMP-2 production between BMSCs by ASCs when grouped by multiplicity of infection and cell passage. At MOI 3/3 (p5), 2 BMSC samples and 30 ASC samples were available. At MOI 3/3 (p3), 8 BMSC and 4 ASC samples were available. At MOI 5/25 (p5), 3 BMSC and 25 ASC samples were available. Mann–Whitney U test was used to determine statistical significance. ^*∗*^ = statistically significant difference (*p* < 0.05).

Several transduction methods with different multiplicities of infection (MOIs) of GAL4-VP16 and LV-TSTA-BMP-2 were tested to determine an optimal protocol. Across all transduction MOIs, ELISA revealed that transduced ASCs (ASC/LV-BMP-2) produced an average of 179.06 ± 151.79 ng of BMP-2 per 1 × 10^6^ cells/24 h at 2 days after transduction, while BMSCs (BMSC/LV-BMP-2) produced 80.05 ± 27.45 ng (*p* < 0.0001) (Figure 3). When comparing BMP-2 production between ASC/LV-BMP-2 and BMSC/LV-BMP-2 at each MOI, significant differences were observed (Figure 4). At a constant MOI of 3/3, ASC and BMSC cells expanded through three passages demonstrated superior BMP-2 production than those passaged 5 times. However, the greatest BMP-2 production was observed in cells that had been passaged 5 times and were transduced at an MOI of 5/25.

**Figure 5:**
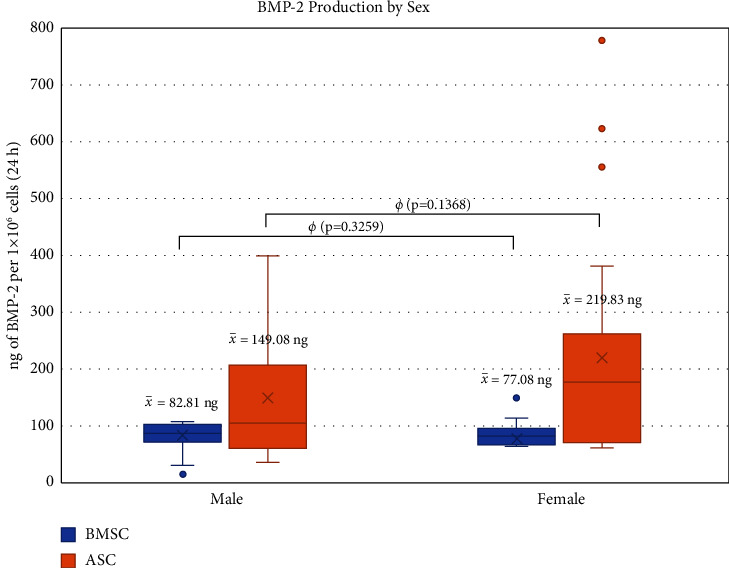
Comparison of BMP-2 production between transduced BMSCs and ASCs when grouped by sex. For male patients, 31 BMSC and 52 ASC samples were analyzed. For female patients, 32 BMSC and 54 ASC samples were analyzed. A chi-squared analysis was used to determine statistical significance. *ø* = statistically non-significant difference (*p* > 0.05).

**Figure 6:**
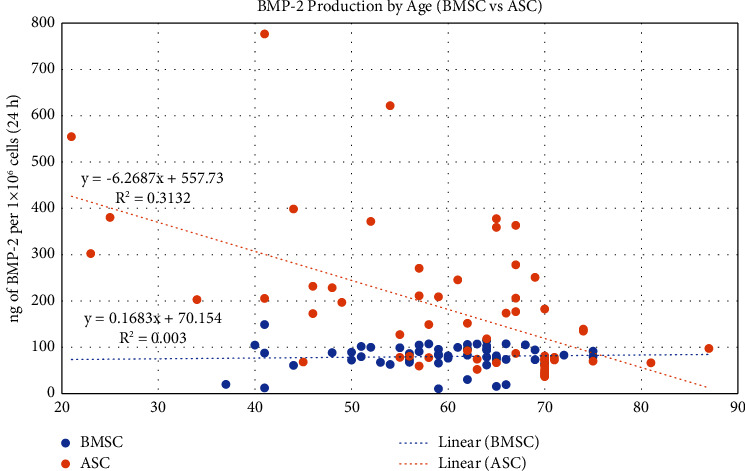
Scatterplot of BMP-2 production by transduced BMSCs and ASCs as a function of age. 63 BMSC samples and 106 ASC samples were included in this analysis. A Pearson correlation analysis was performed to analyze the relationship between BMP-2 production and age.

When assessed by gender, there was no significant difference in BMP-2 production by either ASC/LV-BMP-2 or BMSC/LV-BMP-2 (Figure 5). When plotted by age both continuously and by comparing patients ≤60 years old and those >60 years old, BMP-2 production by BMSCs was not significantly different. However, ASCs harvested from patients >60 years old exhibited decreased BMP-2 production compared to patients ≤60 years old (*p*=0.0007) (Figure 6).

No significant differences in BMP-2 production were noted between transduced ASCs harvested from the infrapatellar fat pad versus lipoaspirate (179.06 ± 151.69 ng versus 88.66 ± 19.69 ng; *p*=0.1627). However, transduced ASCs harvested from AKS demonstrated significantly greater BMP-2 production (381.49 ± 207.91 ng) than cells harvested from TKA (137.74 ± 97.23), due to the younger overall age of the cohort (*p* < 0.0001). A one-way ANOVA indicated significant differences in BMP-2 production following transduction between the TKA, AKS, and lipoaspirate groups (*p* < 0.0001).

## 4. Discussion

The choice of the mesenchymal stem cell population to serve as a cellular delivery vehicle for protein delivery for regional gene therapy for bone repair is usually focused on ASCs and BMSCs. Several factors influence the selection of the cells for potential clinical applications of *ex vivo* gene therapy including the ability to readily harvest these cells from patients, their ability to expand in culture, and transduction efficiency to allow for sustained expression of osteoinductive growth factors. Implementation of a cost-effective and successful regional gene therapy strategy for bone repair requires understanding of the donor-based differences between these cell populations, as well as the factors that affect cell expansion in culture and the BMP-2 production. The primary aim of the present study was to evaluate the differences between human ASCs and BMSCs for potential use in *ex vivo* regional gene therapy focused on upregulating BMP-2. We found that across 187 human subjects, ASCs exhibited more rapid expansion in culture and higher BMP-2 production following LV-TSTA-BMP-2 transduction. Increasing age correlated with diminishing BMP-2 production from transduced ASCs. Furthermore, while ASCs obtained from liposuction had higher SVF and total volume, infrapatellar fat pads produced significantly more BMP-2 after transduction.

Importantly, we examined the ability of stem cells to be expanded in culture and found that ASCs more reliably and quickly expanded in culture when compared to BMSCs. Previous work by Bougioukli et al. [13] investigated the osteogenic potential of BMSCs and ACSs harvested from 26 human subjects transduced with LV-TSTA-BMP-2. The authors noted that ACSs obtained via liposuction had significantly increased BMP-2 production, osteogenic potential, and faster cell growth compared to BMSCs obtained via bone marrow aspirate. The findings of the present study corroborate those of Bougioukli et al. However, the present study assesses a larger patient cohort (187 human donors) including 124 human adipose tissue samples obtained during either arthroscopic or open knee surgery or liposuction. In addition, 63 human bone marrow aspirates were obtained during THA. We demonstrated that while significantly lower initial mononuclear cell counts were noted immediately after harvest in ASCs, the population doubling time of ASCs was nearly one-fourth that of BMSCs with significantly higher cell yields at each passage of growth. Furthermore, of the samples assessed for ability to complete five passages of cell growth, all ASC samples reached P5 while 17.4% of BMSCs were unable to reach P5 due to insufficient growth. Previously, Mohamed-Ahmed et al. examined 9 donor matched samples in pediatric patients and reported that ASCs demonstrated faster proliferation and expandability compared to BMSCs. However, these authors noted significant variability within their smaller cohort of donors [16]. These findings have important relevance in potential clinical applications for *ex vivo* regional gene therapy given the costs and potential patient donor site morbidity associated with stem cell harvest for subsequent implantation.

The BMP-2 production of transduced ASCs when compared to transduced BMSCs demonstrates their potential role in the treatment of bone loss, spinal fusion, and fracture non-union. Several previous studies have investigated the effects of BMP-2 production via LV-TSTA-BMP-2 *ex vivo* gene therapy on osteogenic potential *in vitro* [7, 13, 17]. Variations in mesenchymal stem cell population expansion and pluripotency have been previously attributed to donor characteristics and comorbidities due to underlying physiologic differences [18]. However, Collon et al. [1] demonstrated similar findings within ASC donors with no difference in BMP-2 production or osteogenic potential based on comorbidity burden amongst donor patients. With respect to donor characteristics, we found no significant differences in donor age, BMI, HbA1c, or serum albumin between the BMSC and ASC donors. However, significantly higher BMP-2 production was noted in transduced ASCs compared to transduced BMSCs. Furthermore, ASCs demonstrated significantly higher initial cell yields and faster doubling time in culture compared to BMSCs. These findings may account for the increased BMP-2 production as the ASCs' ability to readily grow and expand in culture may contribute to posttransduction BMP-2 production. In our comparison of cells exhibiting poor versus normal growth in culture, we found no significant differences amongst the BMSC donor characteristics or cell yield. Within the ASC cohort, poor growth was significantly associated with decreased initial SVF and moderately associated with lower serum albumin, though this finding did not reach statistical significance (*p*=0.0561). This suggests that cell density after the ASC harvest and the underlying nutritional status of the patient may be important factors in optimizing growth of human ASCs in culture.

In BMSCs, there was no significant effect of donor age on transduced BMSC BMP-2 production. While ASCs had significantly higher levels of BMP-2 production following transduction compared to BMSCs, we found significantly decreased BMP-2 production in transduced ASCs in patients older than 60 years old compared to younger patients. It has been proposed that BMSC osteogenic differentiation potential and reliability to expand in culture may decrease with age due to epigenetic changes and transcription factor regulation [19, 20]. Similarly, we did not observe sex-based differences in BMP-2 production or cell yield in either BMSC or ASC populations. However, previous studies have noted mixed results with regard to the effect of gender on the osteogenic potential of mesenchymal stem cells [1, 21, 22]. Aksu et al. [21] noted improved osteogenic potential in ASCs harvested from the abdominal wall in male donors compared to female donors. In contrast, Collon et al. [1] found higher ASC yield and cell numbers amongst female patients. Previous in vitro studies using both human and non-human ASCs have shown increased osteogenic potential of samples obtained from males [23–25]. These variations may be explained by transcriptomic level differences which may influence pluripotency, proliferation, and differentiation [22]. More in vitro studies utilizing human BMSCs and ASCs are required to fully elucidate the differences in cell viability and bone regeneration properties amongst a variety of donors.

Both ASC harvesting technique and anatomic location have important clinical implications when considering feasibility and donor site morbidity. We investigated several harvesting techniques across human adipose donors including open and arthroscopic infrapatellar fat pad excision, as well as liposuction. We found that SVF, total tissue volume, and cell number at each passage were significantly higher in the liposuction cohort when compared to the infrapatellar fat pad cohort. However, we found that BMP-2 production following transduction of lipoaspirate was significantly lower than that of both AKS and TKA. Furthermore, human ASCs harvested from AKS displayed higher BMP-2 production despite lower initial SVF when compared to TKA. Several authors have investigated the technique and site variations in cell characteristics in ASCs but have been limited primarily by cohort size [13, 26–28]. A study by Oedayrajsingh-Varma et al. investigated growth characteristics of human ASCs and found that SVF was not affected by the anatomical site of harvest but that direct resection yielded higher frequency of proliferating ASCs compared to lipoaspirate [27]. Contrary to our findings, Iyyanki et al. examined techniques of ASC harvest and found that direct excision yielded higher SVF and total ASCs when compared to liposuction [26]. Furthermore, Schreml et al. compared liposuction to surgical resection and found a higher SVF from liposuction; however, they noted that lipoaspirates could not be differentiated into adipocytes, chondrocytes, or osteoblasts as frequently [28]. This corroborates the findings of our present study in which lipoaspirate yielded higher SVF and improved cellular proliferation. However, once transduced, the lipoaspirate ASCs had lower BMP-2 production compared to infrapatellar fat pad ASCs. These contrasting results in the existing literature highlight the fact that continued investigation of harvest technique and location is required for clinical applications of cell-based therapies. Our results add to the existing body of literature by describing these parameters in the largest study to date including 187 human donors (age range: 21 to 87) with 102 included for final analysis demonstrating improved cell yield and proliferation amongst lipoaspirates compared to excised infrapatellar fat pad samples.

There are several limitations to the present study. First, given the invasive nature of the harvesting procedures undertaken for BMSCs (bone marrow aspiration) and ASCs (lipoaspirate, AKS and TKA), we were unable to compare samples from the same patient. While this limited our ability to control for differences between ASC and BMSC donors, we aimed to mitigate any potential confounding differences with the size of the cohorts and found no significant differences in BMI, hemoglobin A1c, and serum albumin. An additional limitation of the study is the distribution of sample types collected within our study. We were able to collect a total of 106 infrapatellar fat pads (93 from an open approach and 13 from an arthroscopic approach), 18 lipoaspirates, and 63 bone marrow aspirates. This distribution primarily arises from the availability of these samples within clinical practice as the included samples had to be discarded as part of their respective procedures. In most arthroscopic procedures, the fat pad is left intact. Finally, given the retrospective nature of our data, we were unable to perform circular dichroism (CD) analysis of each subpopulation of harvests to further characterize the differences between populations as well as assess stemness of harvested cells. However, the scope of our current investigation aimed at characterizing the differences in growth characteristics and BMP-2 production between BMSCs and ASCs as well as characterizing the differences in ASC harvest site. Finally, we did not evaluate upregulation of chondrogenic or adipogenic growth factors, and thus generalizability of the results of our BMP-2 upregulation in LV-transduced cells to other gene therapy applications should be exercised with caution.

In conclusion, when examining 187 human donors, we found that ASCs demonstrate superior BMP-2 production and cellular proliferation compared to BMSCs. Our results represent the largest human cohort of ASCs and BMSCs evaluated in the literature and greatly expand upon previous studies comparing the potential of ASCs for use in *ex vivo* gene therapy in bone healing [1, 13, 16]. We demonstrated that ASCs harvested from lipoaspirate had higher initial yields and favorable tissue culture expansion compared to infrapatellar fat pad samples, but once transduced with LV-BMP-2 vector, they produced less BMP-2. ACSs remain a leading candidate for autologous cell-based regional gene therapy for bone repair. The results of our study build upon previous investigations to demonstrate the expandability and reliability of transduced ASCs for use in ex vivo gene therapy, as well as their ability to produce osteoinductive growth factors [1, 7, 13]. Future investigations should focus on the patient-related factors that affect *in vivo* bone production associated with transduced human ASCs across various harvest sites.

## Figures and Tables

**Table 1:** Subject demographics.

	Overall cohort (*n* = 169)		Subcohorts				*P* value
			THA (*n* = 63)		TKA/AKS (*n* = 106)		
	Mean	SD	Mean	SD	Mean	SD	
Age	60.61	11.1	59.37	7.57	61.35	12.72	0.166
Sex	86 F, 83 M		32 F, 31 M		54 F, 52 M		**0.018**
Race	114 W, 29 NW, 26 NR		43 W, 15 NW, 5 NR		71 W, 14 NW, 21 NR		
BMI (kg/m^2^)	29.23	6.37	28.37	5.86	29.74	6.62	0.316
Serum albumin	4.31	0.32	4.34	0.33	4.28	0.3	0.187
HbA1c	5.64	0.48	5.72	0.55	5.59	0.43	0.104

THA = total hip arthroplasty, TKA = total knee arthroplasty, AKS = arthroscopic knee surgery, F = female, M = male, W = white, NW = non-white, NR = not reported, BMI = body mass index, HbA1c = hemoglobin A1c, and SD = standard deviation. Bold *p* values indicate significance (*p* < 0.05).

**Table 2:** Cell characteristics.

	THA (*n* = 63)		TKA/AKS (*n* = 106)		*P* value
	Mean	SD	Mean	SD	
Age	59.37	7.57	61.35	12.72	0.166
Tissue volume (mL)	55.56	46.9	10.47	5.13	**<0.001**
Mono/SVF (×10^6^ cells)	246.87	206.82	6.03	6.61	**<0.001**
Cell yield/mL (×10^6^ cells/mL)	8.94	13.23	0.58	0.55	**<0.001**

THA = total hip arthroplasty, TKA = total knee arthroplasty, AKS = arthroscopic knee surgery, Mono = mononuclear cell count, SVF = stromal vascular fraction, and SD = standard deviation. Bold *p* values indicate significance.

**Table 3:** Population doubling times.

	BMSC			ASC (TKA/AKS)			*P* value
	*N*	Mean (days)	SD	*N*	Mean (days)	SD	
P1 to P2	57	45.35	108.88	95	6.14	2.94	**<0.001**
P2 to P3	50	24.67	38.6	76	6.55	3.59	**<0.001**
P3 to P4	44	31.38	28.09	48	8.1	11.8	**<0.001**
P4 to P5	38	23.33	28.26	46	6.93	2.9	**<0.001**

ASC = adipose-derived stem cell, BMSC = bone marrow-derived stem cell, TKA = total knee arthroplasty, AKS = arthroscopic knee surgery, P1 = passage 1, P2 = passage 2, P3 = passage 3, P4 = passage 4, P5 = passage 5, and SD = standard deviation. Bold *p* values indicate significance.

**Table 4:** Comparison of good versus normal growth of BMSCs and ASCs.

	PG		NG		*P* value
	Mean	SD	Mean	SD	
ASC					
*N*	10		95		
Age	57.7	19.13	61.6	11.94	0.359
Sex	6 F, 4 M		47 F, 48 M		0.527
Tissue volume (mL)	8.1	3.57	10.78	5.21	0.116
Mono/SVF (×10^6^ cells)	1.17	1.02	6.49	6.76	**0.015**
Cell yield/mL (×10^6^ cells/mL)	0.16	0.15	0.61	0.53	**0.009**
BMI (kg/m^2^)	31.27	7.34	29.6	6.59	0.455
Serum albumin	4.05	0.36	4.3	0.29	0.056
HbA1c (%)	5.75	0.44	5.56	0.42	0.249
BMSC					
*N*	8		43		
Age	59.37	7.57	59.07	7.29	0.135
Sex	6 F, 2 M		19 F, 24 M		0.109
Tissue volume (mL)	54.38	51.92	58.84	47.73	0.812
Mono/SVF (×10^6^ cells)	193.63	143.05	251.84	223.37	0.483
Cell yield/mL (×10^6^ cells/mL)	5.69	3.98	8.51	13.78	0.572
BMI (kg/m^2^)	25.84	5.8	28.84	5.81	0.310
Serum albumin	4.49	0.32	4.32	0.34	0.241
HbA1c (%)	5.67	0.57	5.77	0.59	0.685

ASC = Adipose-derived stem cell, BMSC = bone marrow-derived stem cell, PG = poor growth, NG = normal growth, F = female, M = male, Mono = mononuclear cell count, SVF = stromal vascular fraction, BMI = body mass index, HbA1c = hemoglobin A1c, and SD = standard deviation. Bold *p* values indicate significance.

**Table 5:** Comparison of adipose harvest site and technique.

TKA versus AKS	TKA (*n* = 93)		AKS (*n* = 13)		*P* value
	Mean	SD	Mean	SD	
Age	64.67	8.27	37.62	14.17	**<0.001**
Sex	47 F, 46 M		7 F, 6 M		0.823
Tissue volume (mL)	11.35	5.35	5.35	3.21	**<0.001**
SVF (×10^6^ cells)	6.7	6.78	1.26	0.95	**<0.001**
Cell yield/mL (×10^6^ cells/mL)	0.57	0.55	0.31	0.33	**0.012**

TKA/AKS versus liposuction	TKA/AKS (*n* = 106)		Liposuction (*n* = 18)		*P* value
	Mean	SD	Mean	SD	

Age	61.35	12.72	39.56	8.51	**<0.001**
Sex	54 F, 52 M		18 F, 0 M		**<0.001**
Tissue volume (mL)	10.47	5.13	378.61	269.53	**<0.001**
SVF (×10^6^ cells)	6.03	6.61	51.39	36.95	**<0.001**
Cell yield/mL (×10^6^ cells/mL)	0.58	0.55	0.13	0.04	**<0.001**

TKA = total knee arthroplasty, AKS = arthroscopic knee surgery, F = female, M = male, SVF = stromal vascular fraction, and SD = standard deviation. Bold *p* values indicate significance.

## Data Availability

The data underlying this article cannot be shared publicly due to HIPAA compliance. Limited deidentified data will be shared on reasonable request to the corresponding author.
